# Respiratory viral triggers and pediatric acute necrotizing encephalopathy: a systematic review and meta-analysis of risk, clinical phenotype, and outcomes

**DOI:** 10.3389/fneur.2026.1839145

**Published:** 2026-10-16

**Authors:** Zhou Liang, Fei Long, Fengli Zheng, Zhi Jun Tan

**Affiliations:** Department of Pediatrics, Guigang People's Hospital, Guigang, China

**Keywords:** acute necrotizing encephalopathy, COVID-19, influenza virus, pediatric encephalopathy, respiratory viral infections, SARS-CoV-2, viral encephalopathy

## Abstract

**Background:**

Acute necrotizing encephalopathy is a rare but severe pediatric neurological disorder characterized by rapid neurological deterioration and characteristic bilateral thalamic lesions on neuroimaging. Increasing evidence suggests that respiratory viral infections, particularly influenza viruses and SARS-CoV-2, play a major role in triggering ANE. However, clinical evidence remains fragmented due to the rarity of the disease. This study aimed to systematically synthesize current evidence on respiratory viral triggers, clinical presentation, neuroimaging features, treatment strategies, and outcomes of pediatric ANE.

**Methods:**

A systematic review and meta-analysis were conducted following PRISMA 2020 guidelines. Electronic databases including PubMed/MEDLINE, Scopus, Web of Science, and Embase were searched for studies published between January 1, 2015, and March 31, 2026 involving pediatric patients (<18 years) diagnosed with ANE associated with respiratory viral infections. Observational studies, case series, and case reports with extractable clinical data were included. Pooled descriptive analyses were performed to summarize demographic characteristics, viral triggers, clinical features, neuroimaging findings, therapeutic interventions, and outcomes.

**Results:**

Fifteen studies comprising 138 pediatric ANE cases were included. Most patients were previously healthy children, predominantly in early childhood. The most common viral triggers were influenza viruses (46.0%) and SARS-CoV-2 (42.3%), accounting for nearly 90% of cases. Clinically, fever (93.4%), altered consciousness (94.9%), and seizures (68.6%) were the most frequent manifestations. Neuroimaging consistently showed bilateral thalamic lesions (94.2%), often accompanied by brainstem (33.6%) or basal ganglia involvement (28.5%). Treatment commonly involved corticosteroids (75.9%) and intravenous immunoglobulin (51.8%), with some patients receiving targeted immunomodulatory therapies such as tocilizumab. Despite aggressive treatment, mortality remained substantial, with 54 deaths among 138 reported cases (39.1%). The random-effects pooled mortality estimate was 39.1% (95% CI: 29.8–49.8%).

**Conclusion:**

Respiratory viral infections—particularly influenza and SARS-CoV-2 are major triggers of pediatric ANE. The disorder is characterized by rapid neurological decline and characteristic bilateral thalamic lesions, with substantial mortality and long-term neurological morbidity. Further multicenter prospective studies are needed to clarify immunopathogenesis and establish standardized therapeutic strategies to improve outcomes.

## 1 Introduction

ANE is a rare and severe form of acute encephalopathy that primarily affects children (1) and is characterized by rapid neurological deterioration and distinctive neuroimaging findings, particularly bilateral symmetric thalamic lesions (2). ANE was initially identified in East Asian pediatric populations and, since then, has been described throughout the world and is progressively being considered as a life-threatening complication of viral infections (3). The condition presents itself clinically with the sudden onset of encephalopathy, which has taken place after a febrile illness. In most cases, this condition is accompanied by seizures, loss of consciousness, and a sudden coma. ANE has remained a disease with high mortality and a significant burden of neurological morbidity in survivors, despite modern medicine in the area of pediatric intensive care and use of immunomodulatory therapy (4). There seems to be mounting evidence to imply that respiratory viral infections are the most frequent precipitating factors of pediatric ANE (5). Historically, influenza viruses are the most common causative agents, especially those that occur during seasonal influenza outbreaks (6). Recently, there have been reports of cases of ANE related to severe acute respiratory syndrome coronavirus 2 (SARS-CoV-2) infection, which has cast new light on respiratory pathogens in the etiology of this syndrome (7–9). There are also other respiratory viruses which are implicated in isolated cases. Despite the fact that the very mechanisms of ANE have not yet been fully elucidated, the existing facts indicate that the disease can be caused by an overreaction of the systemic inflammatory reaction or cytokine storm, which leads to the breakdown of the blood–brain barrier, brain edema development, and necrosis of brain foci (10–12).

The clinical picture of pediatric ANE is extremely diverse, and the results of the reported cases differ dramatically (13). Other children develop and deteriorate neurobiologically very fast and die; other children survive with a certain level of neurobiological disability or, in rare cases, they recover. The possible predictors of disease severity have been suggested as neuroimaging, inflammatory, and host genetic susceptibility factors, especially mutations in the RANBP2 gene (14). Nevertheless, the infrequency of the condition and the predominance of small case series and case reports have constrained the capacity to come up with strong inferences into risk factors, clinical phenotype, treatment, and prognostic outcomes (15). Recent years have witnessed the rise in acknowledgment of the phenomenon of virus-associated pediatric ANE, especially due to the COVID-19 pandemic, which has led to a revival of interest in investigating the connection between respiratory viral infections and this severe neurological syndrome (15). However, the body of evidence is still fragmented, and no overall synthesis of clinical evidence across research studies has been done. To improve the description of the epidemiology, viral triggers, clinical presentation, neuroimaging, therapeutic interventions, and outcomes of pediatric ANE, a systematic review of the available literature is thus necessary.

Hence, the current paper was intended to perform a systematic review and meta-analysis of available clinical literature to assess the importance of respiratory viral infections as causes of pediatric acute necrotizing encephalopathy. In particular, we aimed to analyze the existing evidence in terms of its synthesis concerning the risk factors, clinical presentation, and neuroimaging data, treatment approach, and mortality rate in children with the condition. It is hoped that by incorporating the data of numerous studies, this analysis will help better understand the clinical spectrum of pediatric ANE and guide future research and clinical management of the rare but life-threatening disorder.

## 2 Methods

### 2.1 Study design and reporting guidelines

The study methodology was formulated based on the guidelines of the Preferred Reporting Items for Systematic Reviews and Meta-Analyses (PRISMA) 2020 guidelines, which is a standardized approach to conducting and reporting systematic reviews and meta-analyses. The entire review process, such as literature search, screening, eligibility, data extraction, and synthesis, was conducted as recommended by PRISMA to ensure methodological transparency, reproducibility, and full reporting. The study targeted and reviewed human pediatric populations (<18 years) who had been diagnosed with ANE after respiratory viral infections. Eligible studies were published in peer-reviewed journals and consisted of observational clinical studies, including retrospective cohorts, case series, and case reports. The review protocol was not prospectively registered.

### 2.2 Literature search strategy

A systematic and thorough literature search was undertaken to determine the relevant researches which reported incidences of pediatric acute necrotizing encephalopathy due to respiratory viral infections. Several electronic databases were searched, such as PubMed/MEDLINE, Scopus, Web of Science, and Embase, to cover a wide scope of biomedical and clinical literature. It was intended that the search strategy would include studies published between January 1, 2015, and March 31, 2026, as recent evidence of clinical practice would be captured in those studies, particularly SARS-CoV-2 and influenza outbreaks in particular. Articles published in English only were taken into consideration. Manual screening of reference lists of related articles was also done to find more studies that might not have been obtained via the database search.

A combination of Medical Subject Headings and free-text keywords was used to develop the search terms based on the disease condition, the population, and triggers of the virus. The primary search terms were acute necrotizing encephalopathy, children, and respiratory viral infections. The following representative search strategy was applied: (“acute necrotizing encephalopathy” OR “acute necrotising encephalopathy” OR ANE OR ANEC) AND (child* OR pediatric OR pediatric OR infant OR adolescent) AND (influenza OR “respiratory virus” OR “respiratory viral infection” OR SARS-CoV-2 OR COVID-19). All databases were searched, and the results were imported into reference management software, where duplicate records were identified and deleted before screening. The search syntax was adapted to the indexing system and search interface of each database. Complete database-specific search strategies, including Boolean operators and field specifications, are provided in Supplementary Table S1. Reference lists of eligible studies and relevant articles were additionally screened to identify potentially eligible publications not retrieved through electronic database searching.

### 2.3 Eligibility criteria

There were predetermined eligibility criteria before the screening process to ensure a reliable and objective selection of studies based on the research purpose. These criteria were informed by the population of the study, the interest of exposure, the type of study, and the accessibility of the extractable clinical data.

The studies were included in the study when they dealt with pediatric patients with a diagnosis of acute necrotizing encephalopathy in the setting of respiratory viral infections. ANE was defined using an established clinical-radiological framework consistent with Mizuguchi criteria, requiring acute encephalopathy associated with a febrile/infectious illness and characteristic symmetric bilateral thalamic lesions, with or without additional brain involvement. Studies with insufficient information to verify ANE or distinguish it from alternative encephalopathies were excluded. Some respiratory viral triggers of interest were infections due to influenza viruses, SARS-CoV-2, and other respiratory pathogens that were reported to precede or cause ANE. Original human studies that presented clinical information on pediatric ANE (retrospective cohort studies, prospective observational studies, case series, and case reports) were included in the review. Studies that did not provide extractable data on clinical characteristics, neuroimaging results, treatment methods, or patient outcomes were excluded. Also, peer-reviewed articles published in English in 2015 and 2026 were included to provide up-to-date clinical evidence.

Studies were eliminated if they included animal experiments, mechanistic studies in the laboratory, or non-human research models. Systematic reviews, narrative reviews, meta-analyses, editorials, letters, and commentaries that lacked original patient-level data were also excluded. Abstracts of conferences, meeting proceedings, and reports that were not detailed enough in terms of methodology or clinical details were not deemed eligible. Research that was conducted solely in an adult population was also excluded unless data on pediatrics could be clearly obtained. Besides, those studies where the diagnosis of ANE was not properly delineated or where the appropriate clinical or outcome data were not available were not included in the final analysis. These eligibility criteria were used in the full-text screening phase to make sure that the final dataset contained only those studies that would contain credible and clinically useful information on acute necrotizing encephalopathy in children with respiratory viral infections.

### 2.4 Study selection process

The records, which were found with the help of the database search, were exported into the reference management software, which helped to organize and filter them. Duplications were first identified and eliminated prior to the process of selecting the study. The rest of the articles were screened in two repeated phases, where the title and abstract screening was done, and then the full-text screening. At the first screening phase, two reviewers evaluated the titles and abstracts of all the records that were retrieved to decide their possible relevance to the study objective. At this point, articles that were obviously not in accordance with the predefined eligibility criteria were filtered out. The studies that seemed to be relevant or in whose case eligibility could not be established with reference to the abstract proceeded to the next stage of assessment, the full-text stage.

The second stage involved accessing the full texts of the possibly eligible articles and assessing them individually by the reviewers on the inclusion and exclusion criteria previously established. It ensured that any disagreements or differences between the reviewers, as far as eligibility for the study was concerned is settled by discussing and agreeing with each other. In cases where it was needed, a third reviewer was involved in coming up with a final decision.

### 2.5 Data extraction

Data were extracted using a standardized data-extraction form developed for this review. Extracted variables included first author, publication year, country, study design, sample size, patient age and sex, respiratory viral trigger, comorbidities, reported genetic susceptibility including RANBP2 variants, clinical manifestations, neuroimaging findings, therapeutic interventions, mortality, and neurological outcomes. Data extraction was performed independently by two reviewers, and disagreements were resolved through discussion and consensus, with unresolved discrepancies adjudicated by a third reviewer. Variables not reported in the original studies were recorded as missing rather than considered absent, and no values were imputed for unreported outcomes. For each extracted variable, the denominator was defined according to the number of patients with available information for that specific outcome or characteristic. Missing data were considered as unreported and were not interpreted as absence of the clinical feature, neuroimaging finding, treatment intervention, or outcome.

### 2.6 Methodological quality and risk-of-bias assessment

The methodological quality and risk of bias of the included studies were assessed using the design-specific Joanna Briggs Institute (JBI) Critical Appraisal Checklists. Retrospective cohort studies were evaluated using the JBI Critical Appraisal Checklist for Cohort Studies, case series using the JBI Critical Appraisal Checklist for Case Series, and individual case reports using the JBI Critical Appraisal Checklist for Case Reports. Items were evaluated as “Yes,” “No,” “Unclear,” or “Not applicable,” according to the applicable checklist. The appraisal considered potential biases related to participant selection, ascertainment of ANE and viral exposure, completeness of clinical and outcome reporting, follow-up, and confounding where applicable.

### 2.7 Statistical analysis

Descriptive data were summarized using frequencies and proportions. For outcomes suitable for quantitative synthesis, study-specific proportions with 95% confidence intervals (CIs) were calculated and synthesized using a random-effects model because substantial clinical and methodological heterogeneity was anticipated across studies. Between-study variance (τ^2^) was estimated using the restricted maximum-likelihood (REML) method, and proportions were analyzed on the logit scale and back-transformed for presentation. Statistical heterogeneity was assessed using Cochran’s Q statistic, τ^2^, and the I^2^ statistic, with I^2^ > 50% considered indicative of substantial heterogeneity. Studies reporting zero events were retained in the quantitative synthesis rather than excluded solely on the basis of zero events. Crude aggregate proportions were calculated as the total number of observed events divided by the total number of patients with available outcome data. These descriptive proportions were reported separately from random-effects pooled estimates and were not interpreted as equivalent measures. Because individual case reports may introduce selection and publication bias, a sensitivity analysis excluding case reports was performed for the mortality outcome. The robustness of the mortality estimate was assessed by comparing the primary analysis with the analysis restricted to observational studies and case series. All statistical analyses were performed using R software.

## 3 Results

### 3.1 Study selection

The systematic literature search revealed the existence of 312 records in the chosen electronic databases, such as PubMed/MEDLINE, Scopus, Web of Science, and Embase. Once the duplicate records had been eliminated (a total of 78 records were removed), 234 unique articles remained to be screened by title and abstract. At this step, 192 articles were eliminated due to their irrelevance to the purpose of the study, i.e., pediatric acute necrotizing encephalopathy, adult study samples, review articles or editorials, and the absence of respiratory virus inducements.

The rest of the 42 papers were put through full-text evaluation with regard to eligibility. A total of 27 studies were eliminated for the following reasons: unavailability of extractable pediatric ANE data (*n* = 11), reviews or meta-analyses without patient-based data (*n* = 6), conference abstracts or reports (*n* = 5), and use of non-human experimental models (*n* = 5). In the end, 15 studies (2, 4, 16–28) were included in the final analysis of the study after all predefined inclusion criteria had been satisfied. The studies included were retrospective cohort studies, multicenter case series, and single-case report studies, which reported clinical information on pediatric patients with acute necrotizing encephalopathy after respiratory viral infections. The entire process of work related to study selection, such as identification, screening, eligibility evaluation, and ultimate inclusivity, is summarized in the PRISMA flow chart (Figure 1).

**Figure 1:**
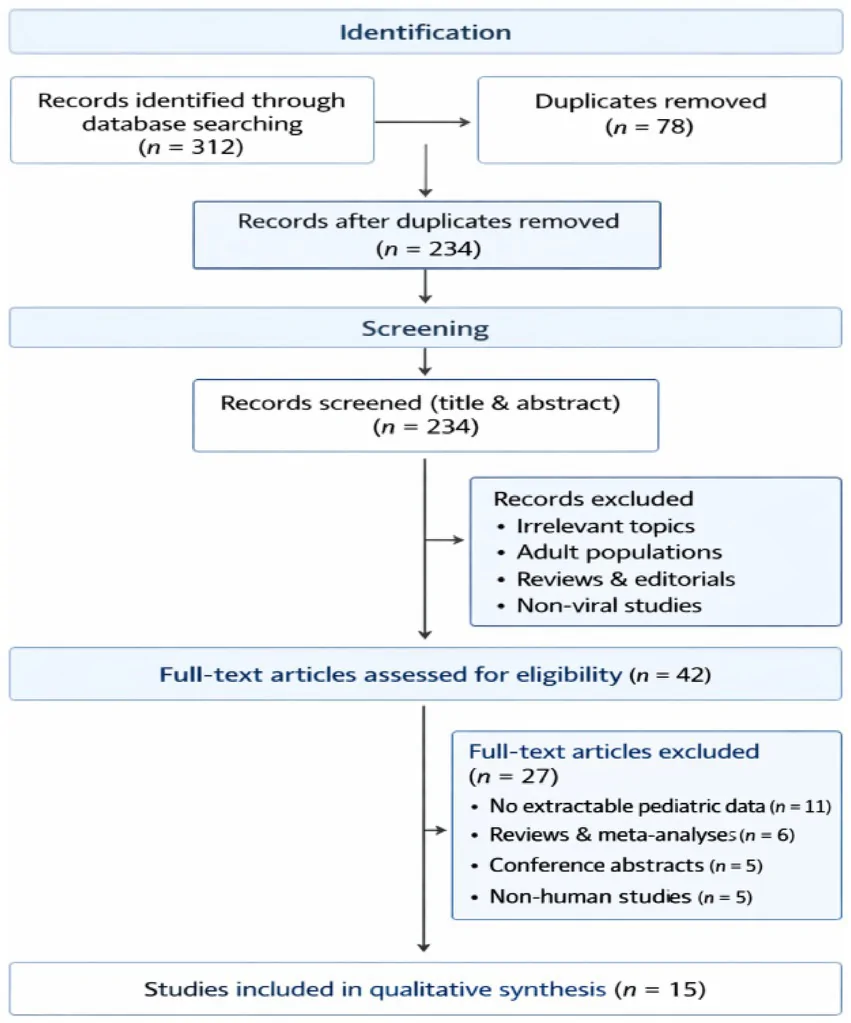
PRISMA study selection flow diagram.

### 3.2 Characteristics of included studies

The 15 studies that underwent the final analysis were written between 2020 and 2025 and presented clinical data on children with acute necrotizing encephalopathy and respiratory viral infections. The articles were a combination of retrospective cohort studies, multicenter case series, and case reports, which is understandable given the low prevalence of this neurological disorder and limited access to big data. In terms of geography, the studies that were included also spread over various regions, such as Asia, North America, and Europe, although some of them were conducted in China as well as in other countries of East Asia, where ANE has been historically more commonly reported. A single multicenter study in the United States provided the highest number of cases of ANE in children with influenza infection. Other literature was based on single-center hospital cohorts or in-depth clinical reports about unique pediatric manifestations.

The sample size across the studies included was quite diverse, with some studies consisting of only one case report, others of multiple centers with a population exceeding 40 pediatric patients. The studies together provided more than 110 cases of pediatric ANE, which provided a wide range of data to analyze the clinical presentation, viral triggers, neuroimaging, treatment strategy, and outcome. The respiratory viral triggers described in the presented studies were related mostly to influenza viruses and SARS-CoV-2, which have been increasingly identified as significant infectious antecedents of pediatric ANE. ANE associated with influenza made up a significant percentage of cases in multiple cohorts, and an increasing number of reports published after 2020 reported COVID-19-associated ANE in children. In certain retrospective cohorts, the triggering agent, virally, was not always conclusively described, but it was preceded by a febrile respiratory illness typical of viral infection. Table 1 summarizes the detailed characteristics of the included studies in terms of study design, geographic location, sample size and viral triggers.

**Table 1:** Characteristics of included studies on pediatric acute necrotizing encephalopathy associated with respiratory viral infections.

Study	Year	Country	Study design	Sample size (ANE cases)	Viral trigger	Key clinical focus
Lin et al. (16)	2023	China	Retrospective case series	12	SARS-CoV-2	Clinical features and mortality of COVID-19–associated ANE
Silverman et al. (4)	2025	United States	Multicenter case series	41	Influenza A/B	Epidemiology, treatment, and outcomes of influenza-associated ANE
Zhang et al. (2)	2024	China	Retrospective cohort	34	Mixed viral triggers	Prognostic factors and mortality predictors in pediatric ANE
Fan et al. (17)	2022	China	Retrospective cohort	12	Viral infection (unspecified)	MRI severity score and neurological outcomes
Chow and Ma (18)	2020	Hong Kong	Retrospective study	8 episodes	Mixed viral triggers	Clinical presentation and prognostic imaging markers
Ma et al. (19)	2024	China	Case series	8	SARS-CoV-2	Treatment outcomes with tocilizumab and immunotherapy
Yue et al. (20)	2024	China	Retrospective cohort	5 ANE deaths	SARS-CoV-2 Omicron	Neurological complications among pediatric COVID-19 cases
Wang et al. (21)	2022	Taiwan	Case report	1	SARS-CoV-2	Neuroimaging and clinical progression
Lazarte-Rantes et al. (28)	2021	Peru	Case report	1	SARS-CoV-2 exposure	Severe neurological progression and imaging findings
Azmi and Abidin (22)	2023	Malaysia	Case report	1	Post-infectious SARS-CoV-2	Clinical recovery after immunotherapy
Forest et al. (23)	2023	Italy	Case report	1	Influenza A / SARS-CoV-2	Recurrent ANE associated with RANBP2 mutation
Zhu et al. (24)	2025	China	Case report	1	SARS-CoV-2	Fatal adolescent ANE case
Largo et al. (25)	2024	Italy	Case series	2	Influenza A	Clinical presentation and treatment outcomes
Jain and Lodha (26)	2020	India	Pediatric neurological series	2 ANE cases	Influenza	Influenza-associated neurological complications
Zhang et al. (27)	2025	China	Retrospective case series	9	Influenza A	Clinical features and outcome predictors

### 3.3 Methodological quality and risk of bias

Methodological quality varied across the included evidence, reflecting the heterogeneous observational and case-based study designs. The evidence base included retrospective cohorts, uncontrolled case series, and multiple individual case reports. The principal methodological concerns were potential selection bias, limited control of confounding, small sample sizes, and incomplete or variable reporting of clinical and outcome data. These concerns were particularly relevant to individual case reports and very small case series, which are inherently susceptible to selection and publication bias and cannot provide reliable population-level frequency estimates. Detailed design-specific JBI assessments are provided in Supplementary Table S2.

### 3.4 Demographic characteristics of the included pediatric population

A total of 138 cases of acute necrotizing encephalopathy caused by respiratory viral infections were found in 15 studies that were included. The pooled population indicated a number of stable patterns in the demographic profile of the population. To start with, the age structure of the cases showed that ANE was mostly among children who were still in early childhood, and yet there were cases across a wide pediatric range (9 months old to 17 years old). Some of the cohort studies reported median ages that clustered around early childhood, as there was a high vulnerability of young patients to severe post-viral neurological complications.

Second, there was a slight female bias in the distribution of sex in the pooled dataset. In general, there were 74 (54.0) females and 63 (46.0) males, which indicates that both sexes have an equal effect but on a small scale, with a female majority in reported cohorts. Third, many percentages of the children affected were reported to be healthy before the development of the neurological symptoms. Through research reporting baseline health status, it was shown that about 76 percent of patients had no recorded neurological or system comorbidity history, which shows that ANE is a common occurrence among healthy children after they have had an acute viral infection.

Fourth, a minority of patients were found to have genetic susceptibility factors, even though most of the cases were sporadic, especially mutations in the RANBP2 gene, which were found to be recurring forms of ANE. These examples indicate the possibility of involvement of host genetic predisposition in disease severity and recurrence. However, all these results prove that pediatric ANE related to respiratory viral infections is a disease of young, previously healthy children with a slightly larger female predominance and genetic predisposition factors that are not very frequent but are significant. Total demographic features of the pooled pediatric population will be presented in Table 2.

**Table 2:** Demographic characteristics of pediatric patients with acute necrotizing encephalopathy.

Variable	Result
Total pediatric ANE cases	138
Age range	9 months – 17 years
Predominant age group	Early childhood (≤6 years)
Female patients	74 (54.0%)
Male patients	63 (46.0%)
Previously healthy patients	~76%
Patients with underlying comorbidities	~24%
Reported genetic susceptibility (RANBP2 mutation)	Rare (<5%)

### 3.5 Respiratory viral triggers of pediatric acute necrotizing encephalopathy

The results of the reviewed literature showed that respiratory viral infections were repeatedly given as the main precipitating factor of pediatric acute necrotizing encephalopathy. In the combined set of 138 pediatric ANE cases, two types of viruses explained the greatest number of reported infections, namely influenza viruses and severe acute respiratory syndrome coronavirus 2 (SARS-CoV-2). The most common viral trigger was influenza viruses, which were documented in 63 cases (45.7%), followed by SARS-CoV-2 in 58 cases (42.0%). Unspecified respiratory viral infections accounted for 17 cases (12.3%). ANE was most commonly associated with influenza A infections, with a small proportion attributed to influenza B infections, which contrasts with large cohort studies, especially the multicenter study of the United States, which helped to illustrate the existing clinical importance of influenza-related neurological complications among children.

The second most prevalent trigger of the infection was SARS-CoV-2 infection, with about 58 cases (42.3%). The prevalence of COVID-19-related ANE reached new peaks after 2020, which is in accordance with the global distribution of SARS-CoV-2 infection and the understanding of the serious neurological effects of the disease. Case series and case reports of rapid neurological deterioration after infection with SARS-CoV-2 had a description of imaging that was consistent with the classical bilateral thalamic lesions of ANE. In another smaller group of cases, the relevant viral precipitant was not conclusively identified. Still, the following neurological syndrome was preceded by a prior febrile respiratory infection, which was suggestive of viral infection. These represented about 16 cases (11.7%) and were grouped as viral infection-associated ANE with unspecified respiratory pathogen. Altogether, the viral trigger distribution suggests that influenza and SARS-CoV-2 were the primary agents in the pathogenesis of ANE in children since they reportedly contributed almost 90 percent to the set of analyzed cases. The frequency of viral triggers in pediatric cases of ANE is shown in Figure 2, and the specific frequency of each viral pathogen is tabulated in Table 3.

**Figure 2:**
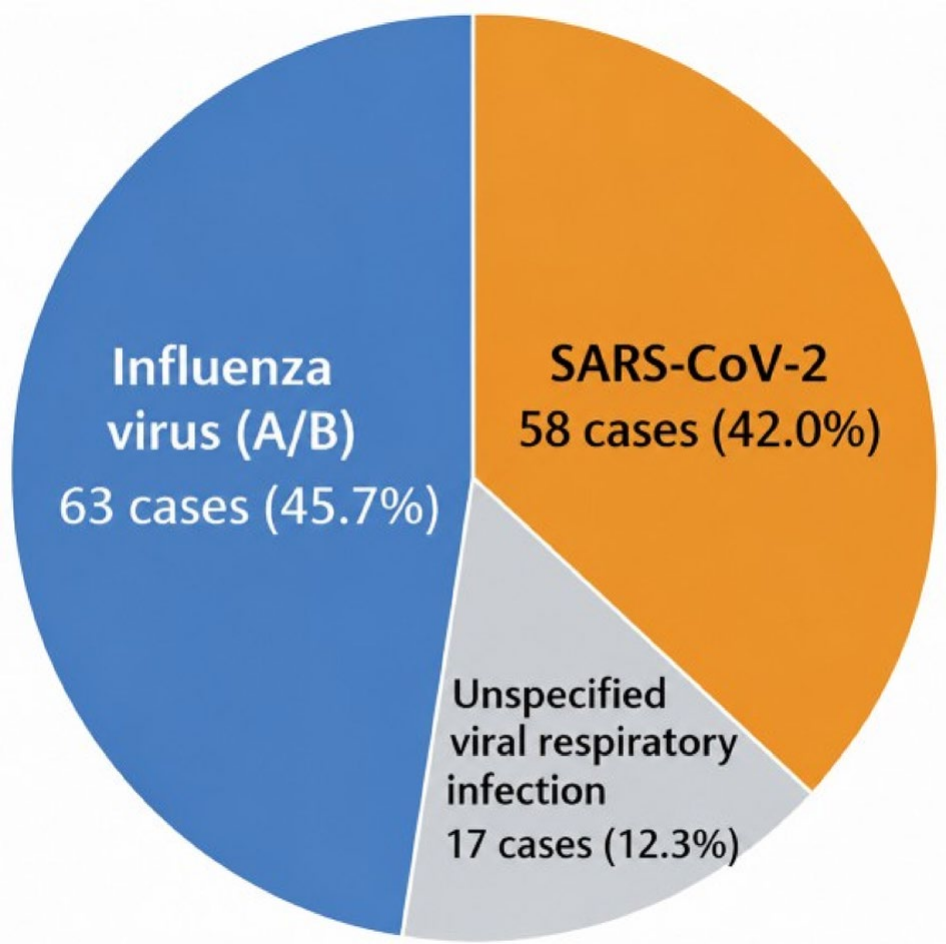
Distribution of respiratory viral triggers in pediatric ANE.

**Table 3:** Distribution of respiratory viral triggers in pediatric acute necrotizing encephalopathy.

Viral trigger	Number of cases	Percentage
Influenza virus (A/B)	63	45.70%
SARS-CoV-2	58	42.00%
Unspecified viral respiratory infection	17	12.30%
Total	138	100%

### 3.6 Clinical presentation of pediatric acute necrotizing encephalopathy

The consistent clinical characteristics of pediatric acute necrotizing encephalopathy in the studies included consisted of a sudden onset of severe neurological symptoms following febrile respiratory illness. The pooled data analysis found a uniform pattern of neurological worsening of the disease, which commonly occurred a few days after the onset of the viral symptoms. The greatest presenting symptom was fever, as 128 of 138 patients (92.8%) reported fever, meaning that an antecedent febrile disease is almost universal in pediatric ANE. There was a high incidence of acute neurological impairment (often after fever), often rapid in progression over a limited clinical period.

Another notable clinical presentation was seizures, which were reported in 94 patients (68.6%). Seizures were the first neurological manifestation in most cases, and a number of reports were of repeated seizures or status epilepticus, indicating severe neurological involvement in the brain. The most recurrent neurological manifestation was altered consciousness or encephalopathy, which was reported in 130 patients (94.9%). The aggressive nature of the disease manifested through the frequent presentation of patients with rapid mental status deterioration, with lethargy, confusion, and deep coma as its end stages.

The systemic manifestations could also be observed in some subset of the patients. Hemodynamic instability or circulatory shock was noted in 21 patients (15.3%), which indicates that the systemic inflammatory response is a possibility in more severe cases. In the same way, 48 patients were undergoing respiratory failure that needed to be supported by mechanical ventilation (35.0%), which reveals the life-threatening state of the majority of affected children and the necessity of intensive care services. In general, the clinical data indicate that ANE in children is usually characterized by fever and acute neurological loss with seizures and impaired consciousness, with systemic complications in severe instances of the disease. Table 4 presents the distributions of clinical features of pediatric ANE in detail.

**Table 4:** Clinical presentation of pediatric acute necrotizing encephalopathy.

Clinical feature	Available data (*n*)	Number of patients	Percentage
Fever	138	128	92.8%
Seizures	136	94	69.1%
Altered consciousness/encephalopathy	136	130	95.6%
Status epilepticus	111	32	28.8%
Vomiting	97	41	42.3%
Circulatory shock	125	21	16.8%
Respiratory failure requiring ventilation	133	48	36.1%

### 3.7 Neuroimaging findings in pediatric acute necrotizing encephalopathy

Findings in neuroimaging were consistently reported in all the studies included and were essential in validating the diagnosis of acute necrotizing encephalopathy. The main diagnostic modality, which was utilized in most of the studies, was magnetic resonance imaging (MRI), which showed a characteristic appearance of symmetric brain lesions, especially of deep brain structures. Bilateral thalamic involvement was the most noticeable and diagnostically characteristic finding and was discovered in 129 of 138 patients (94.9%). These symmetrical thalamic lesions are also well-known as the typical imaging characteristics of ANE and were consistently reported in cases of influenza-associated and SARS-CoV-2-associated encephalitis.

Besides thalamic lesions, brainstem involvement was also reported in numerous studies, and it was found in 46 patients (33.6%). In a number of cohorts, brainstem lesions have been linked to more serious disease and worse neurological prognoses. Lesions of the basal ganglia were seen in 39 (28.5) patients, and those of the cerebellum were seen in 24 (17.5) patients. These data suggest that ANE may have various deep brain locations other than the thalamus, due to the widespread injury to the inflammatory and metabolic capabilities of the disease.

Moreover, 21 patients (15.3%) had hemorrhagic alterations in the lesions, which indicated serious tissue damage and disturbance of the blood–brain barrier. Diffuse cerebral or hemispheric involvement was also mentioned in some of the studies, but this was less commonly described. In general, the neuroimaging findings of the analyzed studies indicate that the bilateral thalamic lesions are the most crucial radiological signs of the ANE in children and that the brain stem, basal ganglia, and cerebellum are also involved in the severity of the disease and prognostic assessment. The frequency of major neuroimaging findings among pediatric ANE cases is summarized in Table 5, while their proportional distribution is illustrated in Figure 3.

**Table 5:** Neuroimaging findings in pediatric acute necrotizing encephalopathy.

Neuroimaging finding	Available data (*n*)	Number of patients	Percentage
Bilateral thalamic lesions	136	129	94.9%
Brainstem involvement	136	46	33.8%
Basal ganglia lesions	136	39	28.7%
Cerebellar lesions	131	24	18.3%
Hemorrhagic lesions	131	21	16.0%
Diffuse cerebral involvement	125	18	14.4%

**Figure 3:**
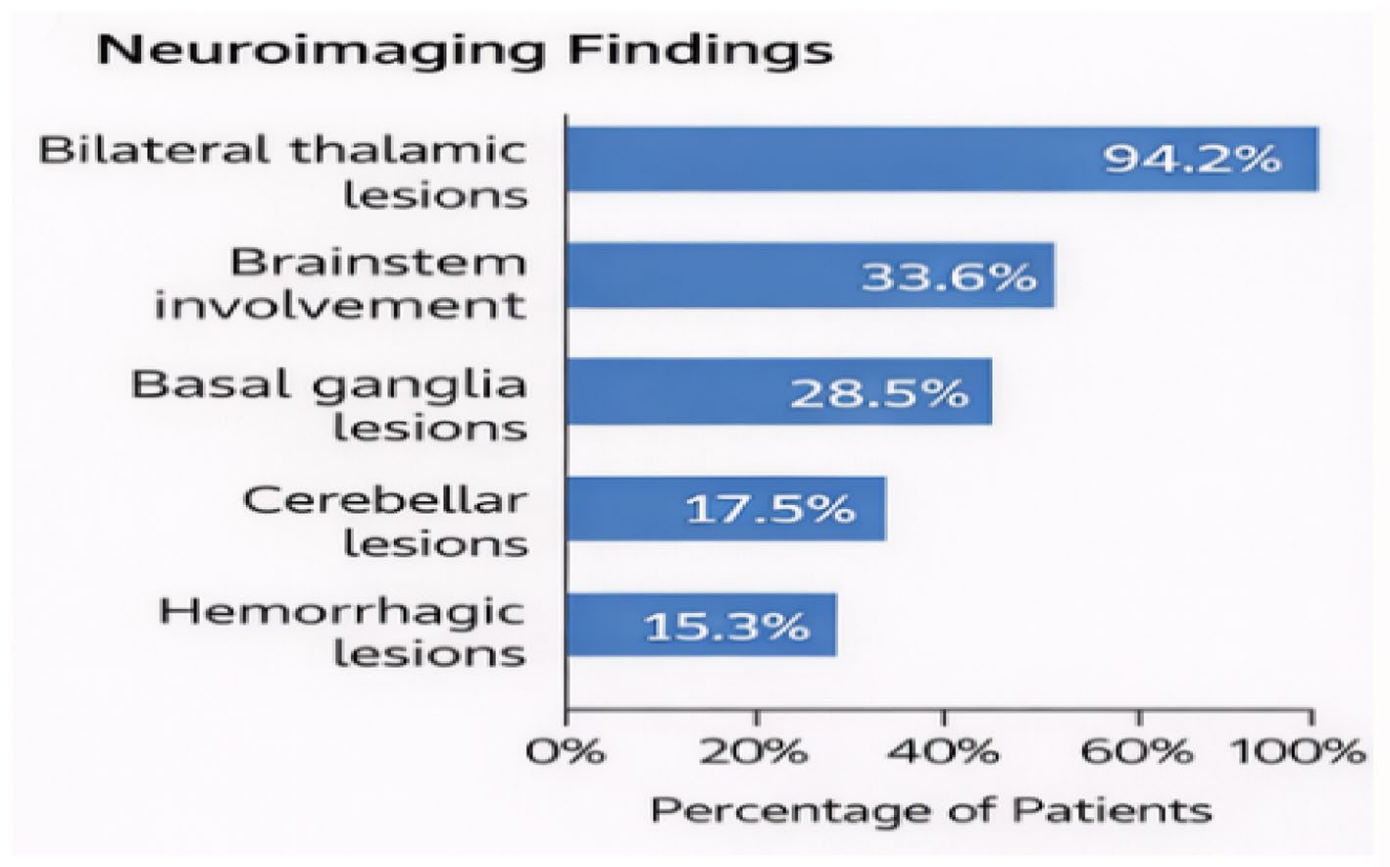
Frequency of neuroimaging findings, highlighting bilateral thalamic lesions as the predominant radiological hallmark, followed by brainstem, basal ganglia, cerebellar, and hemorrhagic involvement.

### 3.8 Therapeutic interventions in pediatric acute necrotizing encephalopathy

The included studies had varied therapeutic management strategies for pediatric acute necrotizing encephalopathy, but all were largely based on early immunomodulatory therapy, with intensive supportive care. Since ANE is thought to be caused by an inflammatory response that is mediated by cytokines, a number of studies have indicated the application of immunosuppressive or anti-inflammatory therapy to restrain neurological damage.

High-dose corticosteroid therapy was the most frequently reported intervention, used in 104 patients with available treatment data. However, these observational data describe treatment patterns and cannot establish therapeutic effectiveness. Some observational studies have suggested potential associations between early immunomodulatory treatment and outcomes; however, these findings remain uncertain because of confounding factors and the absence of controlled comparisons. The second most popular intervention was intravenous immunoglobulin (IVIG), which was employed in 71 patients (51.8%). IVIG was normally administered together with corticosteroids, especially in individuals with rapid progressive neurological deterioration. Most recently, in specific instances, immunomodulatory therapies have been attempted. Responses to tocilizumab, an interleukin-6 receptor inhibitor, were reported among 18 patients (13.1%), especially in cases of SARS-CoV-2-related ANE. Other studies implied possible clinical improvement when IL-6 is inhibited, and the evidence is not very strong because the number of treated patients is insufficient. As a rescue therapy in the worst cases, plasma exchange (PLEX) was administered to eliminate the circulating inflammatory mediators. It has been reported in 12 patients (8.8%), mostly in patients who had refractory disease or severe deterioration of the neurological status.

Intensive care units (ICUs) often had to be supported by the management. The critical state of ANE and the rapid deterioration of neurological dysfunction provided a significant percentage of patients with the need to use mechanical ventilation and hemodynamic support. Generally, the treatment methods of the studies focused on early immunotherapy and aggressive supportive care, yet standardized treatment methods had not been developed, as the condition is rare. The distribution of therapeutic interventions across the included studies is summarized in Table 6.

**Table 6:** Therapeutic interventions in pediatric acute necrotizing encephalopathy.

Treatment strategy	Available data (*n*)	Number of patients	Percentage
Corticosteroids	136	104	76.5%
Intravenous immunoglobulin (IVIG)	136	71	52.2%
Tocilizumab	127	18	14.2%
Plasma exchange (PLEX)	125	12	9.6%
Mechanical ventilation	133	48	36.1%
Intensive care unit admission	134	92	68.7%

### 3.9 Mortality and neurological outcomes in pediatric acute necrotizing encephalopathy

The results of clinical outcomes in the included studies showed that pediatric acute necrotizing encephalopathy is characterized by high mortality and a high risk of neurological disability in survivors. Among the 138 reported pediatric ANE cases, 54 deaths were identified, corresponding to a crude mortality proportion of 39.1%. A separate random-effects meta-analysis was performed to estimate the pooled mortality proportion across studies. Neurological recovery was quite different among the 82 surviving patients. A subgroup of patients attained a positive neurologic outcome, which was survival with a minimum or no chronic neurologic impairments. In total, 28 (20.4% of the entire cohort) patients showed good functional recovery at follow-up. Nevertheless, most survivors had lingering neurological deficiencies, such as motor, cognitive, epileptic, or developmental constraints. Overall, 54 (39.4) patients had survived with moderate to severe neurologic sequelae.

A number of studies demonstrated that mortality was closely linked with a number of clinical and radiological disease severity indicators. Poor outcomes were seen in patients who had brainstem involvement on neuroimaging, a severe change in consciousness, or systemic complications, including shock. Equally, high levels of inflammatory markers and the most extreme cases of metabolic derangements were common in fatal cases in some cohorts. Besides mortality, intensive care support was prevalent in patients with highly diseased conditions. A significant percentage of them needed mechanical ventilation or supportive care, which represents the rapid neurological decline and the systemic inflammatory reaction typical of ANE.

The pooled mortality estimate of the studies is presented in the forest plot (Figure 4), which shows the variation of mortality rates in various cohorts, as well as the high mortality in general in pediatric ANE. Detailed outcome data, including mortality and neurological sequelae, are summarized in Table 7. Sensitivity analysis excluding individual case reports showed comparable mortality findings. After removal of five case-report patients, 52 deaths were observed among 132 patients, corresponding to a crude mortality proportion of 39.4%, which was comparable with the overall mortality estimate of 39.1%. This indicates that the inclusion of case reports did not substantially alter the observed mortality pattern.

**Figure 4:**
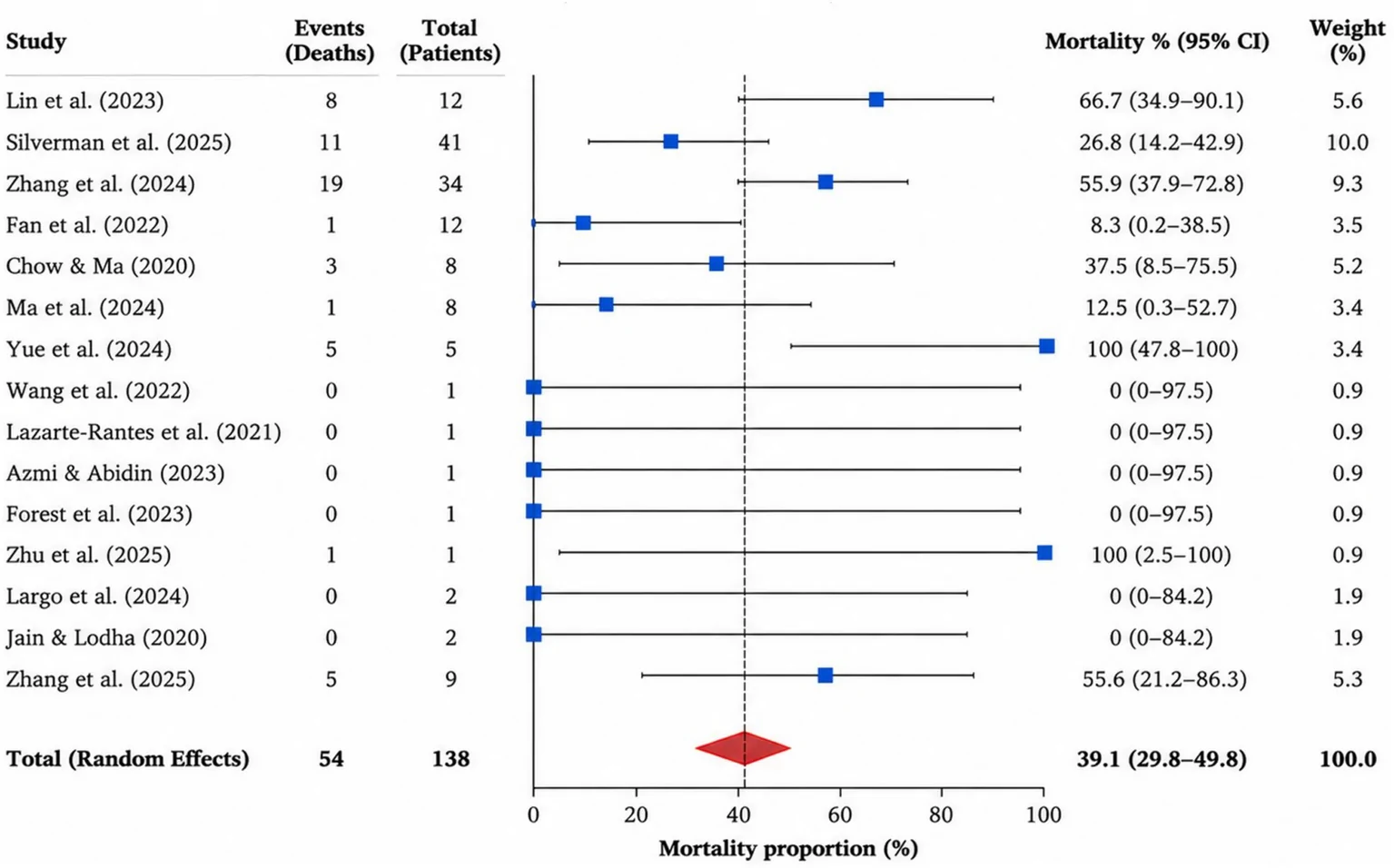
Random-effects meta-analysis of mortality among pediatric patients with respiratory virus-associated acute necrotizing encephalopathy.

**Table 7:** Clinical outcomes of pediatric acute necrotizing encephalopathy.

Outcome	Available data (*n*)	Number of patients	Percentage (Crude)	Pooled estimate (95% CI)
Total ANE cases	138	138	100%	–
Death	138	54	39.1%	39.1% (29.8–49.8)
Survival with good neurological recovery	138	28	20.2%	–
Survival with neurological sequelae	133	54	40.6%	–

Outcome estimates represent reported cases from included studies and should not be interpreted as population-based incidence or prevalence estimates.

## 4 Discussion

Acute necrotizing encephalopathy is a neurological syndrome that is catastrophic but uncommon (mainly affecting children), and is characterized by the rapid worsening of the neurological state in the wake of viral infections (29). In this systematic review and meta-analysis, the contribution of respiratory viral infections as triggers of pediatric ANE has been evaluated, and the current evidence on clinical phenotype, neuroimaging outcome, treatment, and clinical outcome has been synthesized. Beyond confirming established features of ANE, the present synthesis provides an integrated contemporary clinical profile of respiratory virus-associated pediatric ANE across the influenza and COVID-19 eras. Three clinically relevant observations emerge: influenza and SARS-CoV-2 together account for the great majority of reported respiratory viral triggers; acute neurological deterioration accompanied by bilateral thalamic abnormalities represents a recurring clinicoradiological pattern that should prompt early consideration of ANE after a febrile respiratory illness; and the substantial mortality and neurological morbidity observed despite frequent use of immunomodulatory therapy underscore the need for rapid recognition, intensive supportive management, and prospective evaluation of therapeutic strategies.

Among the most interesting results of the study, the significant role of respiratory viral infections in the pathogenesis of ANE in children deserves mention. The most common viral trigger reported in all the studies included was influenza viruses. This is in line with previous epidemiological reports that showed that influenza-related encephalopathy is among the most common causes of serious neurological morbidity in children. ANE was initially described by Mizuguchi et al. as an exclusive clinicoradiological syndrome related to viral infections, especially influenza, that is an example of symmetrical foci of thalamic necrosis and the rapid progression of the neurological condition (30). This relationship has been strengthened by later studies, which have found that influenza A infection is a significant triggering factor in pediatric ANE in seasonal epidemics (31, 32). We thus find that the connection between influenza infection and ANE is well known. Recently, new cases have been reported relating SARS-CoV-2 infection to ANE, bringing a broader range of viral stimuli that cause this syndrome. A number of case reports and small case series have reported children with classical ANE imaging during or soon after infection with COVID-19 (16, 28, 33). These findings are in line with the general neurological complications that have been witnessed during the COVID-19 pandemic. It is believed that the proposed mechanism of SARS-CoV-2-related ANE is immune-mediated inflammatory injury instead of direct viral invasion of the central nervous system, as was previously hypothesized in influenza-associated ANE (34). Our findings suggest that SARS-CoV-2 has emerged as an important reported trigger in the list of triggers of pediatric ANE.

The clinical manifestations in the studies included were relatively homogeneous regardless of the geographical and time variations in viral exposure. The majority of children were first admitted with fever and then tested at a rapid rate with neurological impairment, often with seizures and impaired consciousness. This is the clinical pattern that has been well characterized in past reports of pediatric ANE (30). The acute development of febrile illness to severe encephalopathy is indicative that ANE could be caused by a hyperinflammatory response of the body, typically known as a cytokine storm that causes the impairment of blood–brain barriers, cerebral edema, and necrotising brain injury (35). The findings highlight the role of early diagnosing neurological deterioration among children who present with viral infections. Our study’s neuroimaging results were extremely in line with the radiologic traits of ANE documented in the literature. Symmetrical, bilateral thalamic lesions were identified in the largest possible number of cases, which validates their position as the characteristic imaging changes of this disease. This radiological appearance has been recurrently reported in previous neuroimaging research, and it is deemed to be a diagnostic feature of ANE (30). There was also further activity in the brainstem, basal ganglia, and cerebellum in many cases, and this showed more extensive cerebral damage in severe disease. Other researchers have proposed that brainstem involvement and hemorrhagic changes can be related to poorer outcomes, as it represents widespread neuronal destruction (36, 37). The observed treatment frequencies should be interpreted as descriptions of clinical practice rather than evidence of treatment efficacy. Because most available studies were retrospective and lacked untreated comparison groups, the relative benefits of individual therapies cannot be determined. However, the majority of the patients were put on immunomodulatory treatment, especially corticosteroids and intravenous immunoglobulin (IVIG). Those therapies are hypothesized to target inflammatory pathways involved in ANE pathogenesis; however, their clinical effectiveness remains uncertain because available evidence is derived mainly from observational studies. Prior clinical trials also report similar treatment methods in which there is an early corticosteroid intervention, which has been linked to better neurological outcomes in some patients (36). Tocilizumab, an interleukin-6 (IL-6) receptor antagonist, has been used as a targeted immunomodulatory therapy in selected cases, particularly those associated with SARS-CoV-2-related hyperinflammatory responses. However, evidence regarding its effectiveness remains limited because available data are derived mainly from small observational studies (16). Despite the positive initial reports, the number of cases treated is so small that it is not possible to draw strong conclusions on the efficacy yet.

Although the treatment of ANE in children has been enhanced in critical care and immunomodulatory therapies, the prognosis of the disease is poor. We have shown in our meta-analysis that there was a high pooled mortality rate and a significant percentage of the survivors had persistent neurological sequelae. These data are similar to the previous reports on ANE mortality of 30–40 percent in children cohorts (30, 35). Moreover, survivors will commonly develop long-term complications such as cognitive, motor, and epileptic problems. Some prognostic variables have been suggested in the past literature, such as a high level of initial neurological impairment, high levels of inflammatory markers, and a high level of brainstem involvement in MRI (36). The other significant element of ANE pathogenesis is the genetic vulnerability of the host. The RANBP2 gene has been linked to mutations in familial or recurrent ANE, implying that genetic predisposition can determine the disease susceptibility and severity (35). In addition, Christou et al. (38) reported SARS-CoV-2-associated ANE in a pediatric patient with an RANBP2 mutation, further supporting a potential interaction between respiratory viral triggers and host genetic susceptibility. Although this report was not included in the quantitative synthesis because sufficient patient-level data could not be reliably extracted, it provides relevant supporting evidence for the genetic susceptibility of pediatric ANE. Genetic testing was not always reported in the studies that we included in our analysis, but the fact that such mutations were present sheds light on the complicated nature of the relationship between viral infection and host immune response in the development of ANE.

There are a number of strengths of this study. It initially offers an extensive synthesis of accessible clinical evidence on respiratory virus-related pediatric ANE, bringing together evidence from various geographic areas and viral epidemics. Second, by integrating viral triggers, presenting features, neuroimaging patterns, treatment practices, and outcomes within the same evidence framework, this review identifies a clinically recognizable high-risk phenotype: a child with a recent febrile respiratory illness who develops abrupt encephalopathy or seizures together with bilateral thalamic abnormalities. This pattern should prompt urgent evaluation for ANE and early escalation of neurological and intensive supportive care, while treatment selection should remain individualized because comparative efficacy data are currently insufficient. Nonetheless, there are a few limitations which are to be taken into account. Because ANE is rare, available evidence predominantly consists of retrospective cohorts, case series, and individual case reports. These designs are vulnerable to selection and publication bias; therefore, pooled estimates should be interpreted as descriptive summaries of reported cases rather than population-level epidemiological frequencies. Although individual case reports were retained because of the rarity of ANE, sensitivity analysis excluding these reports produced comparable mortality findings, suggesting that their inclusion did not materially alter the observed mortality pattern. Moreover, the heterogeneity in the study design, diagnostic criteria and treatment protocols may affect the outcome comparisons. Although standardized clinical-radiological criteria were applied, residual diagnostic heterogeneity across retrospective studies and potential misclassification cannot be completely excluded. Lastly, the majority of the recruited studies were retrospective, and this restricts the establishment of causality between treatment interventions and clinical outcomes.

## 5 Conclusion

This systematic review identifies a consistent contemporary clinicoradiological pattern of respiratory virus-associated pediatric ANE across the influenza and COVID-19 eras. Influenza and SARS-CoV-2 constitute the predominant reported respiratory triggers, while abrupt neurological deterioration after febrile respiratory illness and bilateral thalamic abnormalities represent key features that should raise early clinical suspicion for ANE. The substantial mortality and neurological morbidity observed across reported cases support prompt neuroimaging, close neurological monitoring, and early intensive supportive management when ANE is suspected. Although corticosteroids, IVIG, and other immunomodulatory therapies are frequently used, the available observational evidence does not establish their comparative effectiveness. Prospective multicenter studies using standardized diagnostic criteria and outcome measures are therefore required to determine whether specific therapeutic strategies improve survival and long-term neurological outcomes.

## Data Availability

The original contributions presented in the study are included in the article/Supplementary material, further inquiries can be directed to the corresponding author.
