# Supplementary material for: Respiratory viral triggers and pediatric acute necrotizing encephalopathy: a systematic review and meta-analysis of risk, clinical phenotype, and outcomes
Source: Front Neurol. 2026 Oct 16;17:1839145. doi: 10.3389/fneur.2026.1839145 (PMC13719322; doi:10.3389/fneur.2026.1839145)
Supplement: Supplementary file 1 [file Table_1.DOCX]

**Supplementary Table S1. Complete Database-Specific Search Strategies**

| **Database** | **Search strategy** | **Search period/limits** |
| --- | --- | --- |
| **PubMed/MEDLINE** | ("acute necrotizing encephalopathy"[Title/Abstract] OR "acute necrotising encephalopathy"[Title/Abstract] OR ANE[Title/Abstract] OR ANEC[Title/Abstract]) AND (child*[Title/Abstract] OR pediatric[Title/Abstract] OR paediatric[Title/Abstract] OR infant*[Title/Abstract] OR adolescent*[Title/Abstract]) AND (influenza[Title/Abstract] OR "influenza virus"[Title/Abstract] OR "respiratory virus"[Title/Abstract] OR "respiratory viruses"[Title/Abstract] OR "respiratory viral infection"[Title/Abstract] OR "respiratory viral infections"[Title/Abstract] OR "SARS-CoV-2"[Title/Abstract] OR COVID-19[Title/Abstract]) | January 1, 2015–March 31, 2026; English; human studies |
| **Embase** | ('acute necrotizing encephalopathy':ti,ab OR 'acute necrotising encephalopathy':ti,ab OR ANE:ti,ab OR ANEC:ti,ab) AND (child*:ti,ab OR pediatric:ti,ab OR paediatric:ti,ab OR infant*:ti,ab OR adolescent*:ti,ab) AND (influenza:ti,ab OR 'influenza virus':ti,ab OR 'respiratory virus':ti,ab OR 'respiratory viruses':ti,ab OR 'respiratory viral infection':ti,ab OR 'respiratory viral infections':ti,ab OR 'SARS-CoV-2':ti,ab OR 'COVID-19':ti,ab) | January 1, 2015–March 31, 2026; English; human studies |
| **Scopus** | TITLE-ABS-KEY("acute necrotizing encephalopathy" OR "acute necrotising encephalopathy" OR ANE OR ANEC) AND TITLE-ABS-KEY(child* OR pediatric OR paediatric OR infant* OR adolescent*) AND TITLE-ABS-KEY(influenza OR "influenza virus" OR "respiratory virus" OR "respiratory viruses" OR "respiratory viral infection" OR "respiratory viral infections" OR "SARS-CoV-2" OR "COVID-19") | January 1, 2015–March 31, 2026; English |
| **Web of Science Core Collection** | TS=("acute necrotizing encephalopathy" OR "acute necrotising encephalopathy" OR ANE OR ANEC) AND TS=(child* OR pediatric OR paediatric OR infant* OR adolescent*) AND TS=(influenza OR "influenza virus" OR "respiratory virus" OR "respiratory viruses" OR "respiratory viral infection" OR "respiratory viral infections" OR "SARS-CoV-2" OR "COVID-19") | January 1, 2015–March 31, 2026; English |
| **Manual searching** | Reference lists of eligible studies and relevant articles were manually screened to identify potentially eligible publications not retrieved by the electronic database searches. | Same eligibility period: January 1, 2015–March 31, 2026 |
