# Supplementary material for: Respiratory viral triggers and pediatric acute necrotizing encephalopathy: a systematic review and meta-analysis of risk, clinical phenotype, and outcomes
Source: Front Neurol. 2026 Oct 16;17:1839145. doi: 10.3389/fneur.2026.1839145 (PMC13719322; doi:10.3389/fneur.2026.1839145)
Supplement: Supplementary file 2 [file Table_2.DOCX]

# **Supplementary Table S2:** Risk of Bias Assessment of Included Studies Using Joanna Briggs Institute (JBI) Critical Appraisal Checklists

Abbreviations: Y = Yes; N = No; U = Unclear; NA = Not applicable.

## A. JBI Critical Appraisal Checklist for Cohort Studies

| Study | Q1 | Q2 | Q3 | Q4 | Q5 | Q6 | Q7 | Q8 | Q9 | Overall appraisal |
| --- | --- | --- | --- | --- | --- | --- | --- | --- | --- | --- |
| Zhang et al. (2024) | Y | Y | Y | Y | U | U | Y | Y | Y | Moderate risk |
| Fan et al. (2022) | Y | Y | Y | Y | U | U | Y | Y | Y | Moderate risk |
| Chow & Ma (2020) | Y | Y | Y | Y | U | U | Y | Y | Y | Moderate risk |

## B. JBI Critical Appraisal Checklist for Case Series

| Study | Q1 | Q2 | Q3 | Q4 | Q5 | Q6 | Q7 | Q8 | Q9 | Q10 | Overall appraisal |
| --- | --- | --- | --- | --- | --- | --- | --- | --- | --- | --- | --- |
| Lin et al. (2023) | Y | Y | Y | Y | Y | U | Y | Y | Y | Y | Low–moderate risk |
| Silverman et al. (2025) | Y | Y | Y | Y | Y | U | Y | Y | Y | Y | Low–moderate risk |
| Ma et al. (2024) | Y | Y | Y | Y | Y | U | Y | Y | Y | Y | Low–moderate risk |
| Largo et al. (2024) | Y | Y | Y | Y | U | U | Y | Y | Y | Y | Moderate risk |
| Zhang et al. (2025) | Y | Y | Y | Y | Y | U | Y | Y | Y | Y | Low–moderate risk |

## C. JBI Critical Appraisal Checklist for Case Reports

| Study | Q1 | Q2 | Q3 | Q4 | Q5 | Q6 | Q7 | Q8 | Overall appraisal |
| --- | --- | --- | --- | --- | --- | --- | --- | --- | --- |
| Wang et al. (2022) | Y | Y | Y | Y | Y | U | Y | Y | Moderate risk |
| Lazarte-Rantes et al. (2021) | Y | Y | Y | Y | Y | U | Y | Y | Moderate risk |
| Azmi & Abidin (2023) | Y | Y | Y | Y | Y | U | Y | Y | Moderate risk |
| Forest et al. (2023) | Y | Y | Y | Y | Y | U | Y | Y | Moderate risk |
| Zhu et al. (2025) | Y | Y | Y | Y | Y | U | Y | Y | Moderate risk |
| Jain & Lodha (2020) | Y | Y | Y | Y | Y | U | Y | Y | Moderate risk |

## D. Summary of Risk of Bias Across Included Studies

| Study Design | Number of Studies | Overall Risk |
| --- | --- | --- |
| Retrospective cohort studies | 3 | Moderate risk |
| Case series | 5 | Low–moderate to moderate risk |
| Case reports | 6 | Moderate risk |

*Note:* Risk of bias was assessed using Joanna Briggs Institute (JBI) Critical Appraisal Checklists according to study design. The included studies were predominantly observational and case-based; therefore, the main sources of potential bias included small sample sizes, lack of control groups, incomplete follow-up, and variable reporting of clinical outcomes.
